# Association of Dental Practice as a Risk Factor in the Development of Carpal Tunnel Syndrome

**Published:** 2013-03

**Authors:** A Borhan Haghighi, H Khosropanah, F Vahidnia, S Esmailzadeh, Z Emami

**Affiliations:** a Dept. of Neurology, School of Medicine, Shiraz University of Medical Science, Shiraz, Iran; b Dept. of Periodontology, School of Dentistry, Shiraz University of Medical Science, Shiraz, Iran; c Dentist

**Keywords:** Carpal Tunnel Syndrome, Electrodiagnostic Testing, Dentist, Work Duration

## Abstract

**Statement of Problem: **Carpal tunnel syndrome (CTS) is an important cause of work disability. There is controversy over the relation between carpal tunnel syndrome and occupation. The aim of this study was to assess the relationship between the time-span of practicing dentistry and the role of dominant hands in the development of carpal tunnel syndrome.

**Materials and Method: **In this descriptive cross sectional study, 40 dentists and dental students (15 women and 25 men) undertook the electroneuro-diagnostic test in both hands by an electromyogram (EMG) and they were also evaluated in terms of self- reported clinical symptoms.

**Results:** 17.5% of participants were diagnosed to have decreased nerve conduction velocity while10% had reported clinical symptoms of CTS. Both dominant and non-dominant hands were involved. Within cases who were diagnosed as having median nerve neuropathy, 87.5% worked more than 20 hours per week. 57% had 17-23 years of dental practice experience and 14.2% of cases had10-16 years of practice in dentistry.

**Conclusion: **The high rate of CTS symptoms, in both dominant and non-dominant hand among dental practitioners with more years of dental practice, indicates a prequisite for particular attention, then sufficient education on the major risk factors causing this problem. Early diagnosis of these symptoms may improve the future management of the disease.

## Introduction

Carpal Tunnel Syndrome (CTS) is one of the nerve-related disorders which mainly involve the median nerve of the wrist and it can be instigated by exposure to vibration. The frequency of CTS is 1 to 2% in general population [1]. Although the clinical diagnosis of CTS is based on the patient’s t medical history and clinical symptoms, electrodiagnostic test adds objective evidence to the confusing clinical representation. Many patients complain of numbness, tingling and pain in the hand which occurs as a result of the vascular and neural problems [2].

Risk factors associated with CTS include mechanical stress, forceful exertion, monotony of the work and posture. Furthermore prolonged closure of the palm, contact stress and flexure of the shoulders during the work are other factors associated with CTS and other musculoskeletal conditions [3]. CTS occur more frequently in women, in white obese people and in patients with diabetes mellitus, rheumatoid arthritis and thyroid disease [4-5].

There is a debate on whether the carpal tunnel syndrome is work-related or not. Falkiner et al. reviewed 64 articles which were conducted on the relationship between CTS and occupation, they found that CTS is highly related to the underlying systemic disease such as diabetes mellitus or osteoarthritis and lifestyle such as obesity, smoking and long time alcohol intake. Meanwhile, occupation was in the bottom of the list of risk factors in the development of CTS [6]. On the contrary, higher incidence of CTS in working population calls for a relation to occupation. Some studies indicated that certain group of occupations, such as those which requires using vibratory instruments, frequent flexion and extension and forceful wrist movements will uphold the occurrence of CTS [7-9].

Recently, Barcenilla et al. in a peer-review study of published articles from 1980-2009, analyzed the CTS risk factors in the exposed and non-exposed workers. They declared that in exposed workers, there was a strong association between vibration (odds ratio5.4), hand force (odds ratio 4.23) and repetition (odds ratio 2.26) with increased jeopardy of CTS [10]. Contemporary dentistry is associated with technological advancements and introduction of new devices and techniques which are believed to have given rise to the new health problems.

Researchers have investigated several diagnostic techniques and criteria to confirm CTS. These include Tinsel’s nerve percussion test, Phalen's Sign, wrist flexion test, reverse Phalen test, Tourniquet test, Tethered median nerve stress test, Carpal compression test and finally, Electro diagnostic test (EDX) as a gold standard examination. Combination of clinical symptoms and electric conductance have been suggested as the most effective approach. This may lead to a reliable diagnosis of CTS among high- risk populations [11-12].

Considering the importance of this pathological condition and its high incidence rate among different groups of healthcare workers explicitly in the field of dentistry, it is necessary to determine the severity of the problem. To the best of our knowledge, there is limited information regarding the relation of CTS and dental work in our community. Hence we attempted to quantify the association of CTS and dental practice by using self- reported symptoms and an objective assessment of the health of the median nerve of the wrist by nerve conduction measures.

## Materials and Method

In this cross-sectional study, forty non-obese (BMI<25) non- smoker dentists and dental students (15 females and 25 males) of Shiraz Dental School with mean age 37.2 and systemically healthy (with no history of the diabetes mellitus, arthritis and thyroid diseases) were recruited in a random pattern after reading and signing the informed consent form. 

They were initially asked to complete a questionnaire to render the following information: age, sex, weight, height, years of working in dentistry, weekly working hours and presence of any symptoms such as pain, tingling or numbness in the hand or fingers during the day and night.

Then they were evaluated in terms of clinical examination and electric conduction velocity in the motor and sensory branches of the median nerve in both hands by an expert neurologist. Nerve Conduction Velocity (NCV) was measured using an electromyogram (EMG). A ring recording electrode was placed on the third digit and two electrical stimulations were made using a superficial electrode at 14 and 7 cm from the digit on the wrist and the palm respectively.

Subjects with over 3.3 ms median sensory peak latency, over 4.4 median motor peak latency and less than 49 NCV were diagnosed with median nerve distal latency [17].

## Results

Of the 40 participants, seven patients (17.5%) were diagnosed with median sensory nerve distal latency, six of them (85.7%) reported to work over 20 hours in the week and only one participant (14.2%) worked less than 20 hours.

 In terms of years of working experience, one case (14.2%) reported 3-9 years, one case (14.2%) reported 10-16 years and 5 cases (71.4%) reported 17-23 years of working experience. Four cases of CTS (57.1%) were symptomatic, all of whom worked over 20 hours in the week. Of the symptomatic cases of CTS, 3 patients (75%) reported to have 17-23 years of working experience and one patient (25%) belonged to the 10-16 years of working experience category.

## Discussion

Carpal tunnel syndrome is a common neuropathic condition which affects the median nerve in 1- 2% of general population. It is clinically diagnosed by pain, tingling and numbness of the thumb, index, middle finger and half of ring fingers and typically worsens at nights and with repetitive activity [3]. Although different tests and methods have been proposed for the diagnosis of CTS, clinical symptoms and electroneurologic evaluation are regarded as the most reliable diagnostic tool [13].

The present cross sectional study evaluated the median nerve neuropathy among 40 dentists and dental students with different ages, years of working experience and working hours per week. A considerable proportion of the participants (17.5%) were diagnosed with median sensory nerve distal latency which was in agreement with Bingham’s reporting in industrial workers, indicated that dental procedure may be as harmful as some industrial occupations [14].

In a study enrolled on dental hygienist had treated more than half of the patients with heavy calculus showed that they had 2.3 times more hand problems than those who had treated patients with less heavy calculus [15]. Meanwhile in a survey in Sweden, only 3% of the dental hygienists were diagnosed with CTS [16]. This may be attributed to better oral health status of the Swedish people and the large number of dental hygienists compared to the number of patients.

In the present study, 71.4% of the cases diagnosed with CTS reported 17-23 years of work in dentistry and 85.7% more than 20 hours per week. The consistent finding revealed that the dental hygienist with more than 10 years practice were 1.9 times more prone to the CTS symptoms [15]. This illustrates that prolonged working periods may be associated with the increased risk of CTS.

In this study, there is no significant difference between the dominant and non-dominant hand in terms of the incidence of CTS. In this regard, some studies have merely evaluated the dominant hand [9, 17]. These findings can be interpreted that, regardless of the type of instrument implied and the hand engaged, the pressure imposed on the non-dominant hand and fingers and the position of the wrist and the palm during retraction of cheek and tongue play an important role in the development of neurologic symptoms.

Concerning the clinical symptoms, 4 cases were diagnosed with symptomatic neuropathy including pain, tingling and numbness of the fingers (10%) whereas other three cases were diagnosed with asymptomatic prolonged nerve conductance. The same percentage (10%) of the cases diagnosed as CTS with clinical symptom was reported in Bingham’s study [14]. Werner et al demonstrated that even after 18 months, people diagnosed with prolonged median sensory nerve conduction were at greater risk of developing CTS symptoms compared to their matched peers with normal nerve conductance [17]. This revealed that prolong nerve conduction velocity can be considered as a subclinical entity preceding any clinical symptoms and can suggest neurological examination as a routine procedure in all dental personnel including dental hygienists, dental students and dentists.

The high rate of CTS symptoms, in both dominant and non-dominant hands among dental practitioners with more years of dental practice, indicates a prerequisite for particular attention, then sufficient education on the major risk factors causing this problem. Early diagnosis of these symptoms may impact the future management of the disease.

## Conclusion

This study demonstrated that median sensory nerve distal latency may affect the relatively high number of dentists but about half of them represented clinical symptoms such as pain, numbness and tingling sensations. Dentists who imparted more years of dental practice were more likely to present the CTS symptoms. Furthermore, considering the role of nerve canal anatomy and its association with median nerve neuropathy, it is suggested that dental students, initially undergo neurological tests and receive the appropriate information and training on how to protect themselves from potential problems in future.
